# Understanding and acceptance of open, laparoscopic, and robotic surgery among nursing students: implications for educational curricula based on a mixed-methods study

**DOI:** 10.1007/s11701-025-02373-7

**Published:** 2025-06-18

**Authors:** Esra Özkan, Askeri Çankaya, Hatice Özsoy

**Affiliations:** 1 Department of Surgical Nursing, Giresun University Faculty of Health Sciences, Giresun, Turkey; 2 Department of Surgical Diseases Nursing, Fırat University Faculty of Health Sciences, Elazığ, Turkey; 3 Gölhisar Vocational School of Health Services First and Emergency Aid Program, Burdur Mehmet Akif Ersoy University, Burdur, Turkey

**Keywords:** Knowledge level, Mixed design research, Nursing student perception, Surgical education

## Abstract

This study aimed to assess university nursing students’ knowledge and perceptions of open, laparoscopic, and robotic surgery applications. A simultaneous sequential nested quantitative–qualitative hybrid research method design was conducted. The sub-sample for the qualitative phase was conducted with 35 nursing students using the maximum variation sampling strategy with 423 nursing students studying at two universities located in the north and south-east of Türkiye. The data for quantitative were collected using a structured questionnaire comprising two parts created by the researchers in two parts to determine the nursing student's knowledge levels about surgical techniques and to determine the students' sociodemographic characteristics. The qualitative part consisted of six open-ended questions to determine the level of knowledge of open robotic and laparoscopic surgery. Descriptive data were assessed using numbers, percentages, mean, and standard deviation for quantitative analyses. Qualitative data were collected using thematic analysis after data saturation was achieved. It was found that 90.5% of nursing students knew about open surgery, 70.7% knew about laparoscopic surgery, and 62.4% were familiar with robotic surgery. Nursing students' knowledge levels regarding open, laparoscopic, and robotic surgery were 5.26 ± 2.531, 2.498 ± 2.498, and 3.69 ± 2.165, respectively. Themes, derived from semi-structured interviews are “safe, effective and patient-centered surgical care optimization supported by technology and artificial intelligence in robotic surgery”, “safe surgical care supported by technology and innovation that increases patient comfort and clinical success in laparoscopic surgery”, “risks, challenges and training requirements for improving patient safety and quality of care in open surgery” were discussed. It reveals the need for more studies comparing the superiority of surgical methods in a clinical context. Enhancing knowledge of surgical techniques can improve nursing students' readiness for modern surgical practices, promoting safer and more effective. Emphasis on technological surgical methods in nursing education and to increase training on the differences in surgical methods**.** The content of the study was explained to all nursing students in the inclusion group, and after obtaining consent from the nursing students who would participate voluntarily, the study was started with the nursing students who participated voluntarily.

## Introduction

Following the development of laparoscopic and minimally invasive surgical techniques, robotic surgery has taken its place among the latest technological advancements in surgical practice [1]. Recognized as one of the most innovative developments in the field, robotic surgery has become increasingly widespread and is now utilized across nearly all surgical specialties [2] Robotic-assisted surgeries are particularly prevalent in cardiothoracic, head and neck, urology, gynecology, and general surgery [3]. The emergence of laparoscopic techniques marked a significant milestone in the history of surgical innovation and paved the way for the development and adoption of minimally invasive surgery (MIS) [4]. These advancements reflect the broader integration of sophisticated technologies into contemporary surgical practices.

Minimally invasive surgery offers numerous advantages over traditional open surgery. These benefits include smaller incisions, reduced risk of infection, less postoperative discomfort, shorter hospital stays, and faster recovery, making MIS the preferred surgical approach in many clinical settings worldwide [5] Additionally, many surgeons report that robotic surgical platforms provide enhanced dexterity, improved ergonomics, and superior visualization compared to conventional laparoscopic systems, which makes robotic surgery easier to perform for a variety of procedures [6–8]. Despite the increasing prevalence of robotic surgery, structured and comprehensive education on the subject remains limited, particularly at the undergraduate level. A study examining the knowledge of nursing students revealed that although 84.3% expressed interest in technology and 79.3% were familiar with the term "robotic surgery," 73% reported having only partially sufficient knowledge of robotic surgery and its related nursing roles whose are responsible for preoperative patient preparation, intraoperative support including positioning and system troubleshooting, and postoperative care, all while ensuring patient safety and adapting to the technological aspects of robotic-assisted surgery [9]. In a similar vein, only 22.6% of medical students reported any prior experience or exposure to robotic surgery [10]. Furthermore, only 44% of nursing students and 41.4% of medical students indicated that their undergraduate curricula included a course covering robotic surgery [11]. These findings highlight a persistent gap in formal training programs, with many students unaware of the indications and technological developments related to robotic surgery [11, 12]

A review of the literature reveals that while the knowledge and interest of medical students regarding robotic surgery have been relatively well studied [10, 13, 14], research focusing on nursing students remains limited [9]. This gap is significant, considering that nursing students, as future healthcare professionals, play crucial roles throughout the surgical process. Their responsibilities extend beyond observation and include preoperative patient preparation, intraoperative support, maintenance of a sterile environment, and postoperative care—such as pain management, infection prevention, and patient education [15, 16]. These roles differ fundamentally from those of medical students and necessitate a deep understanding of surgical techniques and perioperative care.

In light of the limited number of qualitative studies involving nursing students, this study aims to explore nursing students’ perceptions of robotic surgery. In today’s healthcare landscape, rapid technological advancements are transforming the delivery of medical services, and nurses play a central role in adapting to these innovations. Aligning nursing education with digital health practices and advanced technologies is therefore essential for preparing future healthcare professionals. Nursing students’ awareness and attitudes toward such technologies are not only crucial for their individual professional development but also for integrating technological innovations into healthcare systems and enhancing the quality of patient care. Thus, the findings of this study will provide valuable insights into the future of nursing education and contribute to shaping healthcare services in a more effective, safe, and sustainable manner in line with technological progress.

## Methods

In this mixed-methods study, which explored nursing students’ understanding and acceptance of open, laparoscopic, and robotic surgery, quantitative and qualitative data were integrated using a nested and explanatory sequential design. The initial phase involved a structured survey to quantitatively assess nursing students’ knowledge levels, attitudes, and perceived readiness regarding the three surgical approaches. This enabled the identification of general trends and statistically significant differences among subgroups. Following the quantitative analysis, qualitative interviews were conducted with a purposively selected group of nursing students to explore the underlying reasons behind the observed patterns and to deepen the understanding of their perceptions. For instance, in cases where nursing students reported low confidence in robotic surgery, the interviews provided insights into the contributing factors—such as limited clinical exposure, curricular deficiencies, or uncertainty regarding technological competencies. Qualitative findings were used to elaborate on and explain the quantitative results, thereby offering a richer and more nuanced interpretation of the data. Furthermore, triangulation was applied by comparing responses across both data sets. Areas of convergence helped strengthen the credibility of the findings, while divergences revealed critical contextual and experiential insights that might have been missed using a single method alone. This integrated approach not only enhanced the validity and depth of the study but also informed practical recommendations for nursing education curricula, particularly in preparing nursing students for rapidly evolving, technology-driven surgical environments. Quantitative data were collected through a cross-sectional survey, while qualitative insights were obtained via open-ended questions designed to explore participants’ perceptions and knowledge regarding open, laparoscopic, and robotic surgical techniques. This integration not only enhanced the validity and depth of the study but also informed practical recommendations for nursing education curricula, particularly in terms of preparing students for technologically evolving surgical environments. The study adopted a Concurrent Sequential Nested Quantitative–Qualitative Research Design, as suggested by Creswell (2003). In this study, a Concurrent Sequential Nested Quantitative–Qualitative hybrid research design was preferred. This method was structured to meet the research's goal of revealing general trends and relationships through numerical data in large sample groups, as well as the need to deeply understand the meanings, experiences, and contextual dynamics behind these data. Quantitative data were used to determine the scope, prevalence, or patterns of a specific phenomenon as the basis of the study. In this way, it was possible to reach generalizable results and increase the objectivity of the research. In line with these obtained data, a qualitative data collection process was planned with a smaller number of participants; thus, explanatory, in-depth and contextual information was obtained for certain findings. Throughout the research process, the authors followed the Consolidated Criteria for Reporting Qualitative Research (COREQ) for qualitative reporting (Tong et al., 2007) (see Table 1), and applied the Strengthening the Reporting of Observational Studies in Epidemiology (STROBE) guidelines for the quantitative component. Especially thanks to the "nested" structure, the qualitative data collection process was designed to be derived from the quantitative stage and embedded in it. Thanks to this integrated approach, answers were sought not only to the question "what is happening?" but also to the questions "why is it happening?" and "how is it experienced?". This methodological integrity not only increases the validity and reliability of the research but also enables the multidimensional analysis of complex social and professional phenomena. In this way, it is aimed that the research findings will contribute to both theoretical knowledge production and practical recommendations.

**Table 1:** Consolidated criteria for reporting qualitative research (COREQ)

No.	Item	Guiding questions	Explanations
Personal Characteristics			
1	Interviewer/ facilitator	Which author/authors conducted the interview or focus group?	The first and second author conducted the interview
2	Credentials	What were the researcher's credentials?	First author PhD Second author PhD Third author PhD
3	Profession	What was their occupation at the time of the study?	First author Dr Surgical Nursing Second author Dr Surgical Nursing Third author Dr Surgical Nursing
4	Gender	Was the investigator a man or a woman?	First author Woman Second author Male Third author Woman
5	Experience and training	What experience or training did the researcher have?	The first author has taken courses on qualitative research Second author: has taken qualitative courses and has experience in qualitative research. The third author has taken courses on qualitative research
Relationship with participants			
6	Relationship status	Was a relationship established before the training started?	No relationship was established before the start of the study
7	Interviewer'sparticipant information	What did the participants know about the researcher?	The individuals knew that the researcher had a doctorate in the field of surgical diseases nursing
8	Interviewer characteristics	What characteristics were reported about the interviewer/facilitator?	At the beginning of each interview, the participants were informed about the purpose and objectives of the study
Area 2. Study design			
9	Methodological orientation and Theory	Which methodological orientation was specified to support the study, e.g., discourse analysis, ethnography, phenomenology, content analysis?	This study is a quantitative and qualitative study based on volunteerism
Participant selection			
10	Sampling	How were the participants selected?	In the qualitative phase, the sub-sample group was selected with the maximum variation sampling strategy. Based on the students' responses to the survey, students, consisting of five students from the second-, third-, and fourth-year nursing departments with the most extreme or different beliefs, were purposefully considered. One student
			who did not want to participate in the study was excluded from the study. The qualitative section was completed with a total of … students
11	Approach method	How were the participants approached?	The timing of the interviews was determined by the individuals who voluntarily agreed to participate in the study
12	Sample size	How many participants were there in the study?	A total ofindividuals were included in the study
13	Non-participation	How many people refused to participate or dropped out? Reasons?	The individual who refused to participate in the study was 1 student. He was not included because he stated that he did not want to take part in a scientific study
Setting			
14	The setting of data collection	Where was the data collected?	Detailed information is given in the data collection section of the research
15	Presence of non-participants	Was anyone else present apart from the participants and the researchers?	There were no observers
16	Description of the sample	What are the important characteristics of the sample?	Individuals who agreed to participate in the study were included in the study
Data collection			
17	Interview guide	Were questions, prompts and guidelines provided by the authors? Has it been pilot tested?	Detailed information is given in the Methods section
18	Repeat interviews	Have there been re-interviews? If yes, how many?	No
19	Audio/visual recording	Was audio recording or visual recording used to collect data in the research?	Interviews were conducted face-to-face and transcribed on paper
20	Field notes	Were field notes taken during and/or after the interview or focus group?	The responses of all individuals and the researcher's observations were recorded
21	Duration	How long were the interviews or focus groups?	Each interview lasted between 60 min
22	Data saturation	Has data saturation been discussed?	Data saturation is discussed
23	Transcripts returned	Have transcripts been returned to participants for comments and/or corrections?	No

Area 1: Research team and reflexivity

### Sample and inclusion/exclusion criteria and statistical analyses

The study was conducted with nursing students during the 2023–2024 academic year. The population consisted of nursing students from the Health Sciences Faculties (*N* = 1000) at two universities located in northern and southeastern Türkiye.

The sample included second-, third-, and fourth-year nursing students who voluntarily agreed to participate (*N* = 423). First-year nursing students were excluded, as they had not yet completed the surgical nursing course required for informed participation. This census-style approach ensured that all eligible nursing students, specifically those who were active in their educational progression and had completed the necessary coursework, were invited to participate. Randomization was applied in qualitative section, as the selection was based on voluntary participation from eligible nursing students who met the required criteria. This approach enabled a focused analysis of the knowledge and attitudes of nursing students actively engaged in their education, supporting the development of targeted curriculum and educational strategies. To ensure an adequate sample size, a minimum of 300 participants was calculated using the Raosoft sample size calculator with a 95% confidence interval and a 5% margin of error. However, to minimize missing data and enhance the reliability of the findings, all eligible nursing students were invited to participate, resulting in 423 voluntary participants. Quantitative data were analyzed using SPSS 24.0 (Statistical Package for Social Sciences), with descriptive statistics including frequencies, percentages, means, and standard deviations. For qualitative data, thematic analysis was employed once data saturation was achieved. The failure of new themes to emerge in the last few interviews constituted data saturation. In forming the qualitative study group, a maximum variation strategy was employed among the 423 participants who took part in the quantitative phase. Participants were ranked from highest to lowest based on their total scores, and the top 15 and bottom 15 scoring nursing students were selected for the qualitative interviews. This approach ensured the inclusion of diverse perspectives and varying levels of understanding regarding the study topic. Although data saturation was achieved after 30 interviews, additional 5 participants were included using the same selection criteria to enhance the diversity and reliability of the data and qualitative data were analyzed manually.

### Qualitative research team and reflexivity

The research team comprised two active faculty members from nursing schools, each holding a doctorate in surgical nursing. All members had prior experience as clinical nurses and were trained in qualitative research methodologies.

The study group was identified through criterion sampling, a purposive sampling technique involving the selection of individuals, events, or situations that meet specific predefined criteria relevant to the research problem [18]. Semi-structured, in-depth interviews were conducted with second-, third-, and fourth-year nursing students enrolled in the Faculty of Health Sciences at universities located in two provinces of northern and southeastern Türkiye. Interviews were conducted until data repetition occurred, and data saturation was reached after interviewing 35 participants. Beyond reaching saturation, participant diversity was intentionally prioritized to include nursing students with varying levels of clinical experience, academic performance, and interest in surgical nursing. This approach allowed the study to capture a broad range of perspectives related to the understanding and acceptance of open, laparoscopic, and robotic surgery among nursing students. Consequently, the findings offer more robust insights for the development of responsive and tailored educational curriculum recommendations.

### Inclusion criteria for research

- To be studying in the nursing program,
- To be studying in the second, third, and fourth years
- To have taken surgical nursing course
- To be open to communication,
- Voluntary participation in the research.

### Criteria for exclusion from research

- Having a language, speech, or hearing impairment that prevents communication,
- To be studying in a program other than nursing program,
- Be a first year nursing student.

### Quantitative and qualitative research data collection tools

In the study, "Introductory Information Form" consists of questions about the demographic characteristics of the nursing students. In the data collection form prepared with literature [1], 5-item questionnaire including demographic data of student nurses (Age, Gender, University, Graduated High School, and Classroom) and also quantitative data were collected through a 10-question information form, which assessed the nursing students' knowledge of open, laparoscopic, and robotic surgery. Each correct answer was evaluated as 1 point, and wrong or blank answers were evaluated as 0 points. According to the level of knowledge, the scores were classified as low (0–3), medium (4–7), and high (8–10) knowledge level. The total score obtained indicates the level of knowledge; a higher score indicates a higher level of knowledge. The form was submitted to 3 experts for content validity and the content validity index (CVI) was calculated as 0.92 by expert consensus. Qualitative data form was used "Semi-structured Interview Form" including interview questions to determine the experiences of the nursing students. The supportive sub-questions used in the semi-structured interview were developed in advance, based on the study objectives and existing literature [1]. These questions were included in the interview guide to ensure consistency across participants while still allowing for open-ended exploration.

The interviews were conducted in an environment where only the participant and the researcher were present after the written and verbal consent of the people participating in the research was obtained, with a seating arrangement at an angle of ninety degrees, with the help of a semi-structured interview form in the form of active listening, using the rapid note-taking technique. During the interview, the information, verbal and written consent of the participant was transcribed and the interview was recorded.

### Introductory information form

Introductory information form was prepared by the researcher from the literature [1]. The questionnaire was prepared in line with the questionnaire and consists of 5 questions (Age, Gender, University, Graduated High School, and Classroom) and also quantitative data were collected through a 10-question information form, which assessed the nursing students' knowledge of open, laparoscopic, and robotic surgery.

#### Semi-structured ınterview form

In line with the purpose of the research, the form was prepared from the literature on the subject [1]. The semi-structured interview form was prepared by utilizing the semi-structured interview form consists of supportive side questions to examine the main issues when deemed necessary (the participant keeping silent, going off topic, etc.). The questions in the form are presented below;

1. Which of robot-assisted surgery, laparoscopic surgery, and open surgery techniques do you prefer?

Why? Which factors affect these preferences?

2. Which of the robot-assisted surgery, laparoscopic surgery, and open surgery techniques is more reliable? Which technique do you find more reliable and why?

3. Do these three practices make a difference in nursing practice? Why?

4. What do you think is the effect of these surgical techniques on patient satisfaction and what factors affect this situation?

5. Which of these three surgical techniques attracts you and why?

6. What do you think should be the training to be given to nursing students in robotic surgery?

### Validity and reliability of the study

The four basic criteria of Lincon and Guba (1985) were taken into consideration to try to keep the credibility level of the research high. To see whether the research meets the criteria of credibility (credibility), the results obtained as a result of the analysis of the interviews will be shared with two of the participants with a high level of education and participant approval was obtained. To reach the transferability criterion, i.e., the findings obtained can be used in the other contexts, detailed information about the characteristics of the participants and the place where the research was conducted was provided. In this respect, it is thought that other researchers can reach similar results with similar participants in similar environments. To reach the dependability criterion, detailed information about the research process was presented. In line with the confirmability criteria, in the study results, quotations from the participants' own statements were given before the analyses [17] In order to increase internal validity, opinions were obtained from four experts in their fields for the research questions in the semi-structured interview form and from one expert for the results [17].

### Analyze of qualitative data

The qualitative data obtained from the interviews were analyzed using Colaizzi's (1978) seven-step analysis method for phenomenological studies [18]. Initially, the interview transcripts were read independently and repeatedly by three researchers to gain a thorough understanding of the content. To ensure reliability in the qualitative data analysis process, three researchers independently coded a subset of the transcripts. Following initial coding, joint coding sessions were held to compare interpretations, resolve discrepancies, and refine the coding framework. The researchers discussed differences until a full consensus was reached. Although intercoder agreement was not quantified statistically, thematic consistency was confirmed through iterative discussion and consensus-building.

Significant statements from the texts were identified, reorganized, and expressed in generalized terms. These statements were then analyzed to uncover the underlying meanings. The researchers collaboratively formulated and refined the meanings through discussion until consensus was reached. Subsequently, themes were identified and structured into main and sub-themes, ensuring clarity in their articulation. To enhance the trustworthiness of the analysis, participants' direct quotes were incorporated, allowing readers to verify the interpretation and conclusions drawn from the data [19].

### Ethical approvement

The study received ethical approval from the Giresun University Scientific Research and Publication Ethics Committee (Date: 04/04/2024, No: 04/018, E-50288587-050.01.04-5621). Prior to data collection, informed consent was obtained from all participants. All questionnaires, records, and transcripts were securely stored on a password-protected device. The research adhered to the ethical principles outlined in the Declaration of Helsinki and complied with the standards set by the National Research Committee.

## Results

The mean age of the nursing students participating in the study was 22.66 ± 1.56 years. Among the participants, 70.4% were female, 57% were nursing students from Fırat University, 79.7% were graduates of Anatolian High Schools, and 42.2% were in their second year at university. Descriptive characteristics of the nursing students are presented in Tables 1 and 2.

**Table 2:** Data on the descriptive characteristics of the students

Features	X ± SD	
Age	22.66 ± 1.56 (min19-max34)	
	n	%
Gender		
Female	298	70.4
Male	125	29.6
University		
A University	182	43.0
B University	241	57.0
Graduated high school		
Anatolian high school	337	79.7
Health vocational high school	39	9.2
Science high school	47	11.1
Classroom		
First class	151	35.7
Second class	178	42.1
Third class	94	22.2

It was found that 90.5% of the nursing students were familiar with the principles and techniques of open surgery, 70.7% had knowledge about laparoscopic surgery, and 62.4% were aware of robotic surgery. Additionally, 59.8% of the nursing students reported being able to distinguish between open and laparoscopic surgery, 55.3% could differentiate between open and robotic surgery, and 44.7% could distinguish between laparoscopic and robotic surgery (Table 2).

Moreover, 56.7% of the nursing students stated that they were not familiar with the advantages and disadvantages of laparoscopic and robotic surgeries compared to open surgery. However, 51.1% knew that laparoscopic surgery requires more advanced skills than open surgery, and 50.6% were aware that robotic surgery involves the surgical procedure being performed by a robotic system (Table 3).

**Table 3:** Students' data on open, laparoscopic, and robotic surgery

Questions		n	%
I know what open surgery is	Yes	383	90.5
	No	40	9.5
I know what laparoscopic surgery is	Yes	299	70.7
	No	124	29.3
I know what robotic surgery is	Yes	264	62,4
	No	159	37.6
I know the difference between open surgery and laparoscopic surgery	Yes	253	59.8
	No	170	40.2
I know the difference between open surgery and robotic surgery	Yes	234	55.3
	No	189	44.7
I know the difference between laparoscopic surgery and robotic surgery	Yes	187	44.2
	No	236	55.8
I know the advantage or disadvantage of laparoscopic and robotic surgery compared to open surgery	Yes	183	43.3
	No	240	56.7
Laparoscopic surgery requires more skill than open surgery	Yes	216	51.1
	No	207	48.9
Robotic surgery requires more skill than laparoscopic surgery	Yes	178	42,1
	No	245	57.9
In robotic surgery, the surgical procedure is performed by a robot	Yes	209	49.4
	No	214	50.6

The nursing students' knowledge levels regarding different surgical techniques are summarized in Table 4. The mean knowledge score for open surgery was 5.26 ± 2.531, for laparoscopic surgery 2.498 ± 2.498, and for robotic surgery 3.69 ± 2.165.

**Table 4:** Students' level of knowledge about open, laparoscopic, and robotic surgery

Features	Group	*n*	%	*X* ± SD
Open surgery knowledge level (0–10)	0–3	102	24.1	5.26 ± 2.53
	4–7	236	55.8	
	8–10	85	20.1	
Laparoscopic surgery knowledge level (0–10)	0–3	150	35.5	2.49 ± 2.49
	4–7	236	55,8	
	8–10	37	8.7	
Robotic surgery knowledge level (0–10)	0–3	189	44.7	3.69 ± 2.16
	4–7	218	51.5	
	8–10	16	3.8	

Based on the analysis of the data obtained from the semi-structured interviews, various themes, sub-themes, and codes were identified (Table 5). Table 5a–c presents the themes, sub-themes, and corresponding codes related to nursing students’ knowledge and perceptions of robotic, laparoscopic, and open surgery. The findings are elaborated upon under the main thematic headings based on participant responses.

**Table 5:** Perception and knowledge level of students toward open, laparoscopic and robotic surgery procedures theme, subtheme, and codes

Themes	Sub Themes	Codes
(a) Perception and knowledge level regarding robotic surgery procedure		
1. Optimizing safe and effective patient care with **robotic surgery** technology	A. Security	A1. Patient safety A2. Low risk of infection A3. Low level Pain A4. Low error A5. Reliability A6. Short operation time A.7. Low complication rate
	B. Aesthetics	B1. Aesthetics B2. Small surgical incision
	C. Psychological dimension	C1. Anxiety about the surgical procedure C2. Anxiety about body image C3. Low Trauma
	D. Patient care process optimization	D1. Fast recovery time D2. Short hospitalization period
	E. Operational effectiveness	E1. Mechanical device failure E2. Ease of access to the desired organ or tissue (angular ease)
	F. Technological progress and automation	F1. Resolution and image quality
	G. Cost	G1. Impact on patient budget G2. Impact on hospital budget
2. Safe and effective surgeries with technological progress	A. Operational effectiveness	A1. Rapid Response A2. Rapid discharge A3. Low error rate A4. Safe intervention A5. Fewer device failures A6. Technical problems A7. Ease of access to the desired organ or tissue (angular ease)
	B. Technological progress and automation	B1. Superior software B2. Unmanned application B3. Resolution and image quality
	C. Minimization of complications	C1. Few complications
	D. Hygiene	D1. Sterile
	E. Security	E1. Reliability E2. Low level Pain
3. Optimized patient care with correct resource utilization	A. Patient care process optimization	A1. Easy patient care A2. Rapid dressing A3. Pain monitoring A4. Less pharmacological agents A5. Low complication rate A6. Short hospitalization period
	B. Correct resource utilization	B1. Low use of human labor
	C. Psychological	C1. Rapid psychological impact
	D. Security	D1. Low complication rate
4. The positive effect of technology use on psychological well-being and patient satisfaction	A. Psychological dimension	A1. Trust A2. Psychological resilience A3. Satisfaction A4. High level of comfort A5. Little aesthetic concern
5. Artificial intelligence in robotic surgery the role of innovation and technological developments	A. Innovation	A1. New technological progress A2. Artificial intelligence A3. The idea of a caring robotic nurse A4. Development of software for early fault detection
6. Technical preparation in robotic surgery, the importance of robotic nursing and education	A. Preparation process and technical preparation in robotic surgery	A1. Robotic surgical materials A2. Robot installation
	B. Robotic nursing	B1. Robotic surgery nursing
	C. Education	C1. Robotic surgery course
(b) Perception and knowledge level regarding laparoscopic surgery procedure		
1. Safe patient care with high patient comfort and low aesthetic concerns	A. Security	A1. Low risk of infection A2. Short operation time A3. Reliability A4. Patient safety A5. Low risk of infection A6. Reduced complications A7. Low level Pain
	B. Psychological Dimension	B1. Decrease in body image anxiety
	C. Aesthetics	C1. Aesthetics C2. Small surgical incision
	D. Patient Care Process Optimization	D1. Fast Recovery time D2. Short hospitalization period
	E. Mobilization	E1. Easy movement
	F. Operational Effectiveness	F1. Technical problems
2. Relationship between technology and safe and effective surgeries and clinical success	A. Operational Effectiveness	A1. Trust in technology A2. Rapid discharge A3. Rapid response A4. Anesthesia efficacy A5. Low risk A6. Inability to reach the desired organ or tissue (angular difficulty)
3. Optimized patient care with correct resource utilization	A. Patient Care Process Optimization	A1. Easy patient care A2. Rapid dressing A3. Pain monitoring A4. Late discharge A5. Short hospitalization period
	B. Correct resource utilization	B1. Low use of human labor
	C. Security	C1. Reduced infection rate C2. Low complication rate
4. Strengthening mobilization through psychological support	A. Psychological dimension	A1. Psychological resilience A2. Fear A3. Trust A4. Satisfaction
	B. Mobilization	B1. Easy movement
5. The role of innovation and technological developments in artificial intelligence	A. Innovation	A1. New technological progress A2. Artificial intelligence A3. Surgery without feeling any pain
**6. Technical preparation in laparoscopic surgery, the importance of laparoscopic nursing education**	A. Preparation process and technical preparation in laparoscopic surgery	A1. Laparoscopic surgical materials A2. Technique and application
	B. Education	B1. Laparoscopic nursing course
(c) Perception and Knowledge Level Regarding Open Surgery Procedure		
** 1. Safety** of Open Surgery and functional **challenges** to patient safety	A. Security	A1. Reliability A2. Patient safety A3. Direct intervention A4. Low complication rate
	C. Correct resource utilization	C1. High use of human labor
	D. Aesthetic	D1. Large surgical incision
	E. Mobilization	E1. Limitation of mobility
	F. Quality of life	F1. Restriction in activities of daily living
2. Risks of open surgery	A. Operational Effectiveness	A1. High error rate A2. High rate of complications
	B. Security	B1. Direct intervention
3. Challenges of open surgery and the effects of these challenges on patient care	A. Patient Care Process Optimization	A1. Difficult patient care A2. Wound Management A3.Time Management (More Attention and concentration) A4. Late discharge
	B	B1. High use of human labor
	D	D1. Budget impact
4. Psychological difficulties of open surgery and its effects on postoperative period	A. Psychological dimension	A1. Psychological vulnerability A2. Fear A3. Trust A4. Stress
	B. Operational Efficiency	B1. Rapid Response
5.	–	–
6. The importance of open surgery preparation and training	A. Preparation process and technical preparation in open surgery	A1. Intervention trainings for complications A2. Wound care training

**Main Theme in robotic surgery: "Technology and Artificial Intelligence Supported Safe, Effective and Patient-Oriented Surgical Care Optimisation"** was determined as the main theme. Among the sub- themes, especially safety, aesthetics, psychological dimension, patient care process optimisation, cost, reduction of complications, and correct resource use were determined.

"I would choose robot-assisted surgery because I think that robotic software is less likely to make mistakes in terms of hardware knowledge. The robot can control the complications more than the doctors. Human beings are fallible beings, so I prefer robotic surgery. I think robotic surgery is advantageous in terms of aesthetics. Robotic surgery is also advantageous as a healing process (Ö15)."

"I think robot-assisted surgery is more advantageous than others in terms of aesthetics, recovery, hospitalisation, discharge, infection risk, mobilisation (Ö8)"

"Support should be provided for the application and teaching of robot-assisted surgery. I think that technological approaches also have an impact on the health policies of the country (Ö8)."

“Since today is the age of technology, I believe that training in robotic surgery should be increased. As a result, increasing robotic surgery will turn into daily surgery even in major operations, the discharge of patients will be accelerated, the number of beds and cost will also affect (Ö9)."

**Key theme in laparoscopic surgery: "Technology and Innovation Supported Safe Surgical Care that Improves Patient Comfort and Clinical Success".** Among the sub-themes, especially safety, psychological dimension, aesthetics, mobilisation, patient care process optimization, and innovation were identified. "Laparoscopic surgery seems more reliable. I do not trust robot-assisted surgery because I think there is a possibility that the device may break down. What if it breaks down during the surgical intervention, what will happen then, will my surgery be incomplete? The risk of infection and bleeding in open surgery scares me. Excessive blood loss makes me shudder (S6)."

"Satisfaction is higher in laparoscopic surgery. Because in open surgery, patients have deeper and larger wounds. I also think that the risk of infection is less in laparoscopic surgery compared to others (S8)."

**The main theme in open surgery: "Risks, Challenges and Training Requirements to Improve Patient Safety and Quality of Care"** Among the sub-themes, especially operational efficiency, psychological dimension, preparation process in open surgery, and cost were determined.

"I think open surgery is much safer in terms of patient satisfaction because the perception of robotic surgery has not fully developed in our patients. I think that patients will trust human intervention more, because robotic surgery is a very newly developing field, while laparoscopic is a newer application, so I think that trust will come with time (Ö19)."

"In open surgery, whether the scar will pass or not, the patients' concerns about this issue affect the patient psychology. Robotic surgery is more reliable because the procedure is performed by a robot (S14)."

"There is more pain in patient care in open surgery and laparoscopic surgery. Careful follow-up is required in terms of the wound site, there is a longer healing (Ö14)."

## Discussion

This study aimed to evaluate nursing students’ knowledge and perceptions regarding open, laparoscopic, and robotic surgical methods. The findings highlight students’ awareness and understanding of surgical technologies and offer important insights into the need for integrating technology-based content into nursing education. The rapid advancement of technology in contemporary surgical practice significantly influences perceptions and preferences related to robotic, laparoscopic, and open surgical methods. In this study, nursing students’ views on these surgical approaches were analyzed in relation to various dimensions of technological development and patient care. This study highlighted differences in nursing students’ perceptions of open, laparoscopic, and robotic surgeries. **Open surgery** was seen as familiar and safer, aligning with the high familiarity rate. **Laparoscopic surgery** was viewed as modern but requiring more skill, matching the 51.1% who recognized this need. **Robotic surgery** was praised for precision and safety, though concerns about technical failures and low knowledge levels emerged. Overall, while students had positive attitudes, their knowledge, especially of advanced technologies, was limited, pointing to the need for more technology-focused training in nursing education.

It was determined that the level of knowledge on robotic surgery technique was low. The theme **"Technology and artificial intelligence supported safe, effective and patient-oriented surgical care optimisation"** was evaluated positively in terms of safety, aesthetics, reduced risk of complications, and patient care process optimisation. The perception that robotic surgery minimizes the likelihood of error and can prevent human error emphasizes the role of technology in improving clinical safety. These findings support the literature stating that robotic surgery provides less complications and shorter recovery time with its minimal invasiveness and precision. [20, 21]. However, some nursing students expressed reservations about robotic surgery, especially concerns about the possibility of malfunction of technological devices. This situation shows that more investment should be made in the application and training processes of robotic surgery. In the literature, it is stated that increasing robotic surgery training improves patient outcomes and provides cost-effectiveness [22].

As a result of the analysis of laparoscopic surgery technique, it was determined that the lack of knowledge was at a significant level. It was determined that the prominent themes were "**safe surgical care supported by technology and innovation that increases patient comfort and clinical success"**. The fact that nursing students perceive laparoscopic surgery as a more reliable and satisfaction-oriented method supports the high acceptability of minimally invasive surgeries for patients [23, 24]. However, concerns such as device malfunction compared to robotic surgery have been raised.

It was found that basic surgical procedures were more recognized among nursing students in open surgery. Open surgery technique was discussed within the framework of the theme of **"risks, challenges and educational requirements to improve patient safety and quality of care"**. Especially, disadvantages, such as scars, more pain, and long recovery times, were emphasized by the students. However, a significant proportion of the nursing students stated that they had high confidence in human intervention and found open surgery safer in terms of patient satisfaction. This situation reveals that technology has not yet been fully accepted in patient perception and the need for training in this field [25].

When compared with the quantitative data, it was found that more than half of the nursing students did not have information about the advantages and disadvantages of surgical methods. This suggests that surgical technology should be emphasized more in nursing and medical education. Education of advanced technologies such as robotic surgery may significantly affect the future orientations of nursing care.

The findings of this study align with previous research emphasizing the necessity of enhanced training in advanced surgical methods, particularly robotic and laparoscopic surgeries. Studies by Liu et al. (2024) and Chatterjee et al. (2024) highlight that robotic and laparoscopic surgeries provide fewer complications, shorter recovery times, and greater accuracy, thus enhancing cost-effectiveness in healthcare [20, 22]. These findings highlight the knowledge gaps among students regarding these surgical methods, pointing to the need for further education in these areas. Therefore, integrating advanced surgical technologies more extensively into nursing and medical curricula will better prepare students for the evolving surgical field.

The results of this study indicate a low level of knowledge about advanced surgical methods, especially robotic surgery. To address these knowledge gaps, strategies such as simulation-based training modules should be incorporated. Simulations provide students with a safe and realistic environment to learn robotic and laparoscopic procedures, enhancing their practical experience. Furthermore, it is essential to update curricula to include comprehensive training on robotic surgery and other emerging technologies. Such training would increase nursing and medical professionals' knowledge and skills, ultimately improving patient care safety.

Increased training in advanced surgical techniques will not only raise students' knowledge but also significantly enhance patient safety and care outcomes. Improved education will reduce the likelihood of surgical errors and shorten recovery times. For instance, better knowledge and experience in robotic surgery could help surgeons detect errors more quickly and make better treatment decisions. Additionally, fostering collaborative opportunities between educators and clinical practitioners will help bridge theory and practice, allowing students to face real clinical scenarios more effectively. These collaborations can improve the effectiveness of training in clinical environments.

## Conclusion

It evaluates the effects of surgical methods on patient care process and patient satisfaction from a broad perspective. In addition to the advantages of robotic and laparoscopic surgery, we see that the confidence in open surgery continues. In this context, it can be said that there is a need for more studies comparing the superiority of surgical methods over each other in the clinical context and that there is a need for more emphasis on technological surgical methods in nursing education and to increase training on the differences of surgical methods.

### Limitations

One of the limitations of the study is that all participants were selected from two provinces located in the north and south-east of Türkiye. The results depend on the participants and the environment in which the research was conducted. The study group generally consisted of students with limited experience with these surgical methods. In the region where the research was conducted, robotic surgery is not performed, it is only explained in the training curriculum.

## Data Availability

No datasets were generated or analyzed during the current study.
